# Assessing the Relationship Between Multidimensional Area-Level Indicators and Lupus Disease Activity in Children

**DOI:** 10.3390/children13081062

**Published:** 2026-08-10

**Authors:** Chelsea Reynolds, Paul J. Nietert, Mileka Gilbert, Emily Vara, Natasha Ruth, Joyce Chang

**Affiliations:** 1 Department of Pediatrics, Medical University of South Carolina, Charleston, SC 29425, USA; 2 Department of Pediatrics, Boston Children’s Hospital, Boston, MA 02115, USA

**Keywords:** epidemiology, complications, therapy, statistics, numerical data

## Abstract

**Highlights:**

Prior research suggests that children with systemic lupus erythematosus (cSLE) who live in urban areas with high deprivation and low child opportunity levels are more likely to have severe disease. It is less clear how currently available multidimensional area-level indicators (MDIs) of deprivation, vulnerability, and opportunity perform in mixed rural–urban settings. This study was the first of its kind to assess associations between multiple MDIs and cSLE outcomes in a mixed rural–urban population.

**What are the main findings?**

- Associations were identified between higher social vulnerability and the attainment of a lower lupus disease activity state and lower opportunity levels with the development of central nervous system lupus. However, these findings were not replicated with other indices and should be interpreted with caution. In this predominantly Black, mixed rural–urban cohort, area-level metrics alone did not explain significant differences in severity or disease control.

**What are the implications of the main findings?**

- The available metrics of community-level social conditions may not associate as strongly with disease activity and the development of major organ manifestations of cSLE in a mixed rural–urban setting compared to findings published in urban settings.
- Alternative strategies may be needed to evaluate relationships between neighborhood-level conditions and pediatric health outcomes in mixed rural–urban populations.

**Abstract:**

**Background/Objectives:** Childhood-onset systemic lupus erythematosus (cSLE) is a chronic, multisystem autoimmune disease that is associated with more severe organ involvement, more intensive drug therapy, and increased long-term organ damage compared with adult-onset disease. The objectives of this study were to evaluate the performance of widely used small area-level multidimensional indicators of neighborhood disadvantage in a mixed urban–rural cSLE cohort against disease outcomes. **Methods**: This retrospective cohort study utilized electronic health records to identify and evaluate pediatric patients with cSLE across a single center in South Carolina between 1 January 2020 and 31 December 2024. Primary outcomes included disease activity at diagnosis, measured by Systemic Lupus Erythematosus Disease Activity Index 2000 (SLEDAI-2K); the achievement of a low lupus disease activity state (LLDAS) by the last visit; the development of major organ involvement; and the rates of unplanned hospitalizations and emergency department visits. The associations of the census tract-level Area Deprivation Index (ADI), Social Vulnerability Index (SVI), and Childhood Opportunity Index (COI) with clinical presentation and outcomes were estimated using generalized linear models and logistic regression. **Results:** A total of 85 patients with cSLE were included, of which 76% reported being of Black race, and 28% lived in rural areas. Lupus disease severity was inconsistent across the metrics of area-level social vulnerability, neighborhood deprivation, child opportunity, and rurality. Patients who lived in more socially vulnerable communities were more likely to attain LLDAS during follow-up, while those who lived in lower-opportunity areas were more likely to develop CNS lupus. Baseline disease activity, renal involvement, and healthcare use were not significantly associated with area-level social disadvantages or rurality. **Conclusions:** In this single-center, mixed urban–rural cohort of children with cSLE, indicators of neighborhood-level disadvantage and rurality alone explained little variation in disease severity and disease control overall. The utility of available small area-level metrics is likely context-dependent and influenced by regional features, population characteristics, and the outcomes being studied. Future work should integrate individual- and area-level factors across diverse settings to better understand the drivers of health disparities.

## 1. Introduction

Childhood-onset systemic lupus erythematosus (cSLE) is a severe autoimmune disease associated with substantial morbidity, including increased disease activity, medication burden, organ damage, and complications from therapeutic medications [1]. Lupus in children is often more severe than adult-onset disease, with higher rates of renal and central nervous system involvement and greater healthcare utilization and increased long-term complications including disability, reduced quality of life, and higher mortality [2,3,4,5,6,7]. Although advances in treatment have improved survival and long-term outcomes in cSLE, disparities in disease outcomes endure across racial and socioeconomic groups. These disparities are the most pronounced in historically marginalized populations, including Black, Hispanic, and Native North American communities [4,5,6,7,8]. Systemic lupus erythematosus (SLE) remains the fifth most common cause of nonaccidental death among Black and Hispanic adolescent females [9]. As a result, it is critical to understand the drivers of these disparities in order to inform meaningful interventions to close the gap.

Disparities in cSLE outcomes are shaped not only by individual-level social and biologic factors but also by structural and environmental inequities that influence access to resources and conditions that are important for overall health. For example, for hospitalized patients with cSLE, having public vs. private insurance and living in zip codes with the lowest median household income vs. the highest median income were both significantly associated with an increased hospital length of stay [3]. Extending beyond area-level poverty alone, multidimensional area-level indicators (MDIs), including neighborhood deprivation, social vulnerability, and childhood opportunity, capture a broad range of other social and environmental factors that drive health inequities, including aspects of the built environment, housing, household characteristics, education, and other socioeconomic conditions [10]. These overlapping influences create contexts in which multiple disadvantages can cluster and contribute to increased disease burden and poorer quality of life among children with lupus.

The ability to quantify area-level inequity is vital to understanding and ultimately addressing disparities in cSLE. Currently available MDIs, including the Social Vulnerability Index (SVI), Area Deprivation Index (ADI), and Childhood Opportunity Index (COI), provide standardized methods for measuring community-level disadvantages [11,12,13,14,15,16]. Recent studies suggest that these indices are associated with increased disease activity, the reduced attainment of a low lupus disease activity state (LLDAS), and higher rates of complications and healthcare utilization in cSLE, particularly in urban populations [17,18,19]. In one study evaluating the relationship between ADI and the attainment of LLDAS, a higher ADI was not independently associated with LLDAS after adjustment for renal disease and insurance status; however, renal disease significantly mediated the relationship between the ADI and prednisone exposure [17]. Additionally, this study demonstrated that uninsured and publicly insured patients were more likely to utilize inpatient healthcare resources at diagnosis and reside in neighborhoods with higher pollution exposure and higher ADI scores [17]. Similarly, studies evaluating the COI found that children living in predominantly urban areas with lower opportunity levels had higher odds of severe disease presentation and increased acute care utilization within the first year after the diagnosis of lupus [18,19]. Collectively, these findings suggest that community-level disadvantages may contribute to disparities in cSLE outcomes through multiple pathways, including disease severity, healthcare access, and healthcare utilization. However, existing research has largely focused on urban populations [16,17,18,19]. Consequently, there remains a limited understanding of how specific MDIs are associated with cSLE outcomes in mixed rural–urban populations.

This study aims to evaluate the relationship between MDIs, rurality, and lupus disease activity in children with cSLE in a mixed rural–urban population. Informed by the World Health Organization Social Determinants of Health framework, this study will examine whether composite indicators of higher deprivation, greater social vulnerability, lower childhood opportunity, and increased rurality are associated with worse lupus disease activity, lower attainment of LLDAS, increased organ damage, and greater healthcare utilization [20]. By identifying how area-level inequities contribute to disease burden, this work may help inform targeted interventions, improve resource allocation, and support more equitable approaches to pediatric lupus care.

## 2. Materials and Methods

This was a retrospective cohort study of patients with cSLE who were seen at the Medical University of South Carolina between 1 January 2020 and 31 December 2024. The initial cohort was identified using ICD-10-CM diagnosis codes for SLE (M32.9) associated with a pediatric rheumatology encounter. Exclusion criteria included a diagnosis of discoid lupus erythematosus, neonatal lupus, or overlap syndrome with lupus; a home address outside of the state of South Carolina; or a PO box listed as the home address. The study participants come from 21 of South Carolina’s 46 counties, with 33 (39%) of the 85 study participants coming from the tri-county region (Charleston, Berkeley, Dorchester) surrounding the Medical University of South Carolina (MUSC). Electronic health record data were obtained for each patient who met the inclusion criteria and included sociodemographic factors, medical factors, and lupus-related outcome measures. Sociodemographic factors included age at most recent visit, age at diagnosis, sex assigned at birth, ethnicity, race, and form of insurance. Sex assigned at birth, ethnicity, and race were self-reported at registration. Medical factors of interest included age at diagnosis and severe lupus manifestations, including biopsy-proven lupus nephritis (LN) and the development of central nervous system (CNS) lupus. CNS lupus was defined by clinical documentation that a neurologic manifestation was attributed to active lupus by a rheumatologist. Primary outcome measures included the initial Systemic Lupus Erythematosus Disease Activity Index 2000 (SLEDAI-2K) score and LLDAS attainment at the most recent encounter [21,22]. SLEDAI-2K scores were calculated retrospectively at each encounter within the time period of interest using clinical documentation and laboratory results from each visit. Only visits with adequate clinical and laboratory information needed to calculate all applicable SLEDAI-2K domains were included in this study. The presence of proteinuria was defined by a urine protein-to-creatinine ratio of >0.5. If a diagnosis of SLE occurred before 1 January 2020, the SLEDAI-2K score was calculated at the visit of diagnosis or at the hospital admission associated with the diagnosis. LLDAS was determined by using the clinical encounters with sufficient information required for the calculation of LLDAS [22]. Co-primary outcomes included any lupus-related ED visit or any lupus-related hospitalization. Lupus disease-related ED visits and hospitalizations were identified through chart review, and visits for treatment only or those unrelated to complications from lupus were excluded from this study. This study received an exemption and a waiver of informed consent from the MUSC Institutional Review Board for the secondary use of clinically collected health information.

The primary area-level exposures of interest were census tract-level Centers for Disease Control and Prevention SVI 22, Neighborhood Atlas ADI version 2022, and COI version 3.0, ranked at state levels [12,14,15]. Nationally ranked indices were examined in secondary analyses. These metrics were mapped through the geocoding of street addresses and linked to each patient. The geocoding of addresses was done by linking census tracts or blocks to publicly available, related MDI tools [12,14,15]. Additionally, the rurality of the patients’ residence was examined using a dichotomization of the Rural–Urban Commuting Area (RUCA) code score of the associated 5-digit zip code of residence: a primary RUCA score of less than 4 was considered urban, while a score of 4 or more was considered rural [23]. Descriptive statistics were used to summarize the study population with respect to demographics and clinical characteristics. Continuous variables (i.e., age, SLEDAI-2K scores) were summarized as means, standard deviations, and ranges, while dichotomous outcomes (attainment of LLDAS, development of lupus complications at any time during follow-up [LN, CNS lupus], and occurrence of any SLE-related ED visit or hospitalization during follow-up of 12 months) were expressed as proportions. The proportions of patients achieving dichotomous outcomes were compared between patient subgroups (as defined by SVI, ADI, COI, and rurality).

Next, a series of models was constructed to examine the associations between each of the area-level metrics and clinical outcomes while adjusting for covariates. Essentially, for all models, the outcome was expressed as a function of the MDI of interest while adjusting for a set of pre-specified covariates for confounding control informed by the prior literature and clinical knowledge. No stepwise variable selection method was used. Since the initial SLEDAI-2K score was being treated as a continuous variable, it was modeled as the dependent variable using a generalized linear model (GLM) framework. GLMs are an excellent way of examining the association between two variables while adjusting for covariates. As the MDIs (i.e., the independent variables of interest) are expected to be correlated with each other, separate models were developed for each MDI (to avoid issues associated with multicollinearity). Within the GLM for SLEDAI-2K, we examined whether the mean SLEDAI-2K scores varied across MDI quartiles. Logistic regression models were utilized for all dichotomous outcomes (i.e., the dependent variables). In the logistic regression models, each of the MDIs was dichotomized at its median (to avoid any small cell counts) so that the resulting odds ratios reflected comparisons of higher vulnerability, higher deprivation, and lower opportunity to lower vulnerability, lower deprivation, and higher opportunity, respectively. All multivariable models were adjusted for age, sex, race, ethnicity, insurance type (public vs. private), disease duration, and length of follow-up (LLDAS only). Adjusted odds ratios, their 95% confidence intervals, and two-sided *p*-values were reported. *p*-values < 0.05 were considered statistically significant. No adjustments were made for multiple hypothesis testing. None of the variables of interest were missing in our dataset except for the ADI, which was missing for n = 2 subjects; analyses involving the ADI were conducted on those with non-missing ADI values (n = 83). SAS v9.4 was used for all analyses.

## 3. Results

There were 104 patients who had a diagnosis of SLE who were seen between 1 January 2020 and 31 December 2024. Of these, 19 were excluded (4 had cutaneous lupus, 3 had overlap syndromes, 7 had home addresses not within the state of SC, 2 had a PO box listed for their address, and 3 were subsequently found not to have a diagnosis of lupus). A total of 85 patients with cSLE were included and comprised a predominantly Black (76%) and female (87%) cohort. Approximately half had private insurance (53%) (Table 1). The study population included both rural and urban residency (72% urban vs. 28% rural) (Table 1). Eighty percent of our study participants resided in areas that were above the national median SVI, and 75% lived in areas above the national median ADI. A total of 64% lived in areas designated as having “very low” or “low” opportunity levels relative to the entire U.S. At diagnosis, the patients included in the cohort demonstrated moderate disease activity with a mean SLEDAI-2K score of 14.4 (range of 0–38), which improved over time to a mean score of 5.1 (range of 0–18) over the study period of interest (Table 1). Of the study population, 78% (66 patients) achieved LLDAS by their final visit. LN was the most common complication at 53% (45 patients), followed by CNS involvement (13% or 13 patients) (Table 1).

**Figure 1:**
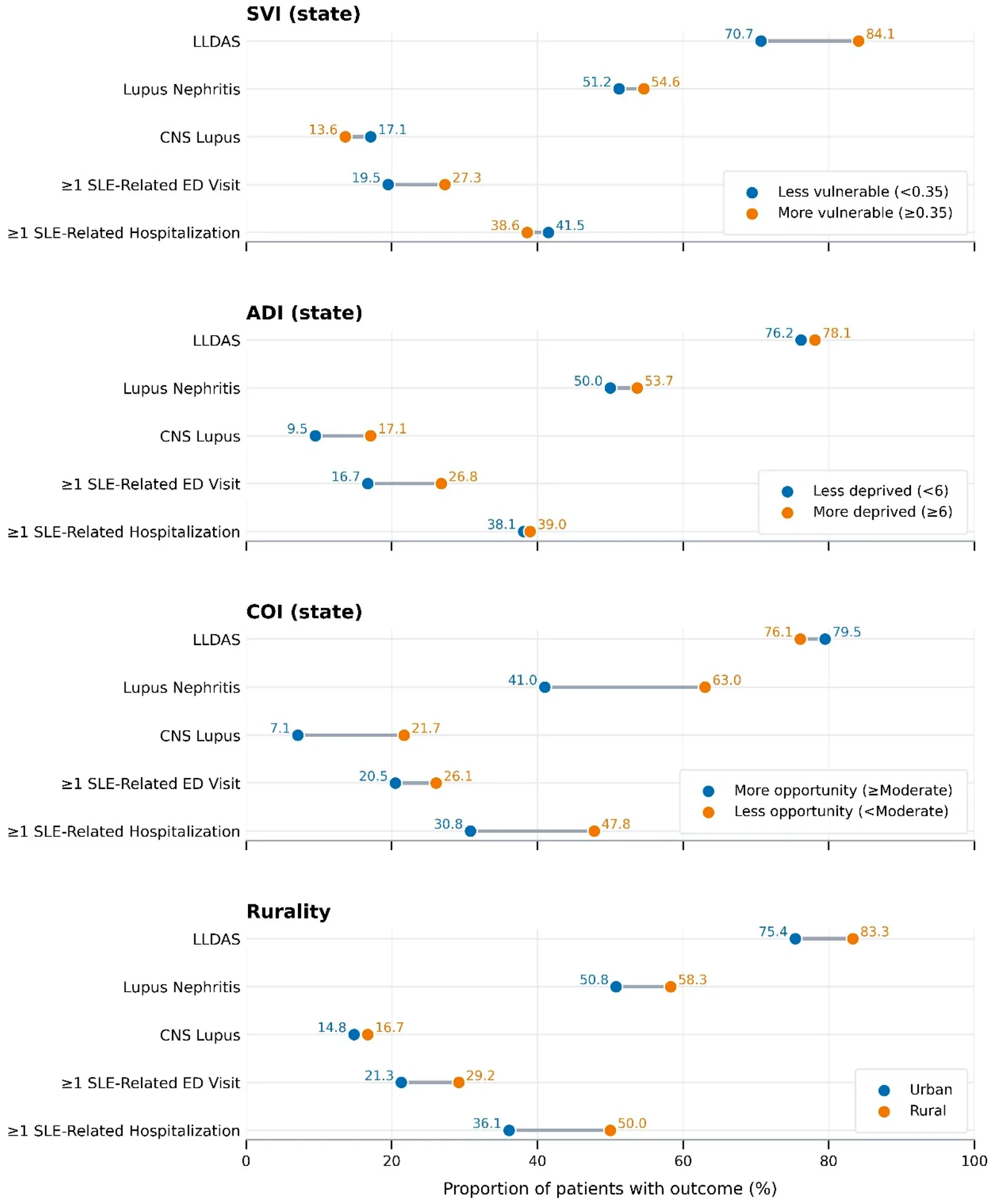
Proportions of patients attaining each outcome, stratified by dichotomizations of state-level SVI, ADI, COI, and rurality.

The unadjusted proportions of outcomes (attainment of LLDAS, organ manifestations) when dichotomizing state-ranked MDIs into low vs. high (opportunity, vulnerability, and deprivation) are shown in Figure 1. Supplemental Table S1 has the raw data presented in Figure 1, along with data for the dichotomized national MDIs. Numerically higher proportions of LN and CNS lupus were observed in higher-deprivation and lower-opportunity areas, with the largest magnitude of difference observed according to COI category (Figure 1). In contrast, minimal to no differences in disease manifestations were observed according to SVI category. With respect to disease control, lower LLDAS achievement was also observed in low- vs. high-opportunity areas, whereas the opposite was observed for the ADI and SVI, with lower LLDAS achievement in lower-deprivation and lower-vulnerability areas, respectively. Secondary analyses stratifying patients based on the national rankings of MDIs yielded similar results (Supplemental Table S1). The results of multivariable logistic regression models comparing binary outcomes by MDIs at the state and national levels are shown in Table 2. These models allowed us to compare outcomes across MDIs while adjusting for relevant covariates (age, sex, race/ethnicity, insurance, duration of disease, and length of follow-up time [LLDAS only]). At the state-ranked level, there was no significant difference in the achievement of LLDAS with respect to social vulnerability. In the secondary analysis of the nationally ranked SVI, there was a statistically significant association between higher social vulnerability and higher odds of achieving LLDAS. However, this was not replicated across other MDIs (Table 2). There was also a statistically significant relationship between the state-ranked COI and CNS lupus, wherein patients living in areas with lower opportunity relative to the state had higher odds of CNS lupus over the study period (adjusted OR 8.70, 95% CI [1.3, 58.0], *p* = 0.026), albeit the level of precision of this estimate was low. A similar direction of effect was observed for nationally ranked COI categories and CNS lupus that was not statistically significant. There were no statistically significant adjusted differences with respect to MDI levels for other outcomes of initial lupus disease activity or LN.

**Figure 2:**
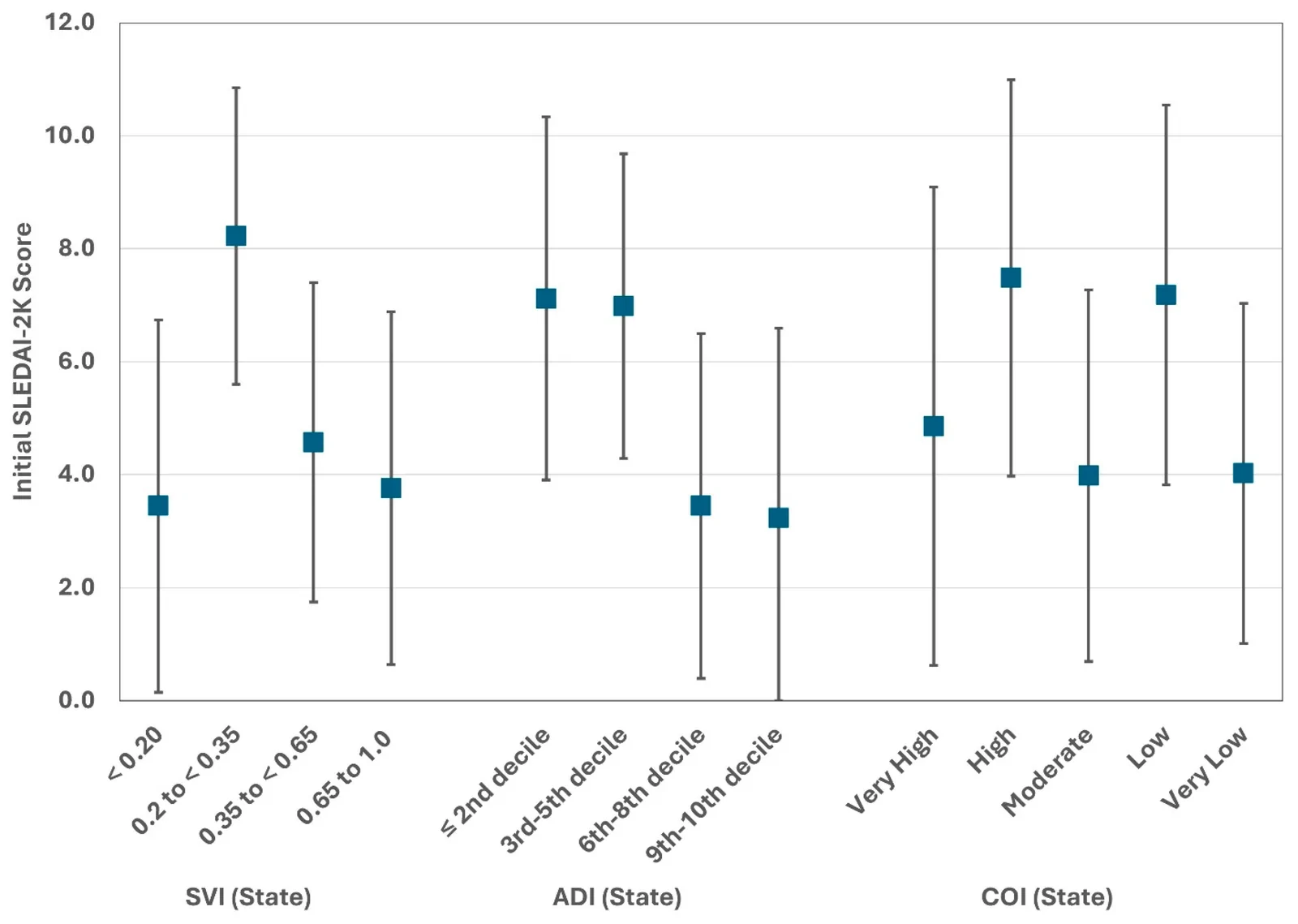
Results of multivariable general linear models comparing initial SLEDAI-2K scores across state MDI categories. Blue squares are model-adjusted least square means, and error bars reflect 95% CIs.

The results of the multivariable general linear models comparing initial SLEDAI-2K scores across state MDI categories are illustrated in Figure 2. Supplemental Table S2 lists more detailed results of the multivariable general linear models comparing initial SLEDAI-2K scores with respect to both state and national MDI categories. Adjusted analyses of MDIs at the state and national levels did not identify consistent relationships across MDI categories with lupus disease activity. There were no significant associations between greater neighborhood-level disadvantages and higher disease activity using any of the three MDIs. Contrary to predictions, patients living in areas categorized as intermediate-vulnerability by the SVI and low-deprivation by the ADI had the highest average disease activity at the last follow-up, adjusted for covariates (Figure 2).

## 4. Discussion

In this cohort of patients with cSLE with high rates of LN, disease severity was similar across communities, regardless of the composite indices of social vulnerability, neighborhood deprivation, opportunity level, or rural residence. Disease activity, major organ manifestations, and healthcare utilization were not strongly patterned by area-level indicators or consistent across the different indicators. Overall, these findings suggest that initial disease activity and the development of complications from cSLE may not be well explained by widely used metrics of community-level social conditions in our mixed rural–urban population.

Our study did not observe robust associations between various MDIs and cSLE disease severity, disease control, or healthcare utilization. This result differs from that of the previously published literature demonstrating strong associations between area deprivation and child opportunity with cSLE outcomes in other cohorts [18]. There are several potential explanations for this observation. First, individual- or household-level resources and conditions may be stronger drivers of cSLE presentation and outcomes than area-level conditions, and the latter is not a proxy for individual-level resources [16]. Second, the variability in the validity and performance of MDIs across both geographical and disease contexts may limit their utility in mixed urban–rural settings. This may be particularly important in contexts where the aggregation of different dimensions of disadvantages over heterogeneous geographic units obscures vulnerabilities in areas where deprivation and rich resources may coexist [23,24]. For example, the Neighborhood Atlas ADI tends to overweight home values, which can significantly underestimate deprivation in urban areas with very high housing costs, such as in Charleston, SC, while overestimating deprivation in rural areas with lower housing costs [24]. Efforts to standardize deprivation factors may improve the sensitivity and specificity of indices such as the ADI [11]. Third, patients in our cohort were concentrated in very-low-opportunity areas relative to both the state and national levels, which may have limited our ability to detect meaningful differences due to a floor effect. Lastly, the construction of these indices may not align with the specific challenges of our region and patient population. For example, patients who travel longer distances from rural areas may depend less on public transit and more on car ownership to access healthcare and would be more likely to be misclassified as less deprived. These issues highlight the key limitations of composite area-level indicators as well as the importance of conducting disease- and setting-specific validations prior to use in health policy or practice.

Despite the lack of robust associations across most outcomes, we did observe that patients in lower-opportunity areas were more likely to develop CNS lupus in both unadjusted and adjusted analyses. Similarly, patients living in more deprived areas as measured by the ADI also had numerically higher rates of CNS lupus, albeit the difference was not statistically significant in adjusted analysis. To our knowledge, while higher rates of LN have been reported to be associated with area-level disadvantages, no literature has reported specifically on the rates of CNS lupus in children in relation to area-level metrics [17]. As our estimates in this single-center cohort were imprecise and may not be generalizable to other contexts, this finding will require further external validation. Although isolated associations were identified, including higher odds of LLDAS attainment among patients from more socially vulnerable communities and lower odds of CNS lupus among those living in higher-opportunity areas, these findings were not replicated across other indices or analytic models and should be interpreted with caution.

Meanwhile, trends toward higher emergency department use and hospitalizations for complications of lupus for patients living in more disadvantaged areas tended to mirror the prior literature [2,3,18]. The unexpected finding that patients living in areas with a higher nationally ranked SVI were significantly more likely to achieve LLDAS during the study period is not well explained by our data. This pattern was not observed for the state-ranked SVI or for ADI or COI. Possible explanations for this observation include residual confounding due to unmeasured individual-level social determinants and differences in access to specialized pediatric care after referral, referral bias, small sample size and subsequent statistical instability, as well as type I error. As a result, this finding should be interpreted with caution.

This study is strengthened by its use of multiple MDIs including the ADI, SVI and COI, which capture distinct yet overlapping constructs. This comprehensive approach enhances the analysis by demonstrating that the observed associations may be index-specific. Prior studies have also noted only a moderate correlation and poor agreement between the SVI and ADI due to the different dimensions of vulnerability that are captured and their relative weights. Taken together with the prior literature, our findings speak to the non-interchangeability and context dependence of these indices. Furthermore, the inclusion of both state- and national-level indices further strengthens interpretation by accounting for differences in geographic scale. Additionally, this study’s focus on meaningful lupus outcomes, such as LLDAS and SLEDAI-2K scores, that are predictive of the long-term accrual of disease damage and mortality increases its relevance to patient care [21,25,26,27,28,29,30]. Notably, this cohort represents a truly mixed rural and urban population, an area that has been underexplored in prior MDI and cSLE research.

We also acknowledge several limitations of this study. This study is limited by its small sample size of patients, which limited our power to detect smaller differences and to compare distinct levels of area-level disadvantage. Due to the small number of patients with CNS lupus, the associated odds ratios had large confidence intervals, indicating imprecision and potential model instability, emphasizing the need for collaborative studies involving larger cohorts. Moreover, this is a context-specific evaluation of patients from a single center, which reduces the generalizability of the disease-specific findings, though the challenges of utilizing MDIs may be more broadly applicable. MDIs assume that all people within a certain area experience similar conditions, but there can be wide variability within a census tract or block, and highly advantaged neighborhoods can coexist within the same area as highly disadvantaged communities. An additional limitation of this study is its reliance on baseline SLEDAI-2K scores and the attainment of LLDAS, as these measures do not adequately capture longitudinal lupus disease trajectories over time. Moreover, the initial SLEDAI-2K was not representative of the SLEDAI-2K at diagnosis for patients diagnosed prior to the study period. The confounding variables of sex, race, ethnicity, and insurance type were considered in the analysis of the data; however, additional confounding variables, including other disease severity indicators and individual-level social determinants (household income, parental education level, etc.), were not considered in the models and represent an additional limitation. Future studies could be done to address these variables and their influence on outcomes in cSLE. The retrospective design, which involved chart review, introduces potential data incompleteness, as any information not documented in clinical notes or results was unavailable for analysis. Future research that includes multicenter studies with patient cohorts from geographically diverse regions, including rural and urban populations, and increases in sample size would improve generalizability. Lastly, additional future research incorporating access to healthcare, including distance to a pediatric rheumatology center, would be valuable to assess the relationship between healthcare access and lupus disease presentation, management, and outcomes.

## 5. Conclusions

As MDIs are increasingly being used to understand the potential influence of structural factors on disparate health outcomes in chronic pediatric conditions such as cSLE, it is critical to understand the context-dependent manner in which they perform. Geographic features, the demographic homogeneity of the neighborhoods to be studied, and the specific outcomes to be measured may all influence predictive validity. In our predominantly Black, mixed urban–rural cSLE cohort concentrated in low-opportunity areas, neighborhood-level metrics alone did not explain substantial variance in major organ manifestations, disease activity, or disease control. Integrating individual- and area-level perspectives will be essential to fully elucidate disparities in childhood lupus and to identify meaningful, context-sensitive targets for intervention. Further research is needed, particularly across diverse geographic settings, to inform targeted interventions and equitable resource allocation to improve outcomes for vulnerable pediatric populations.

## Figures and Tables

**Table 1:** Descriptive statistics for demographic and baseline clinical characteristics.

Patient Characteristics	Summary Statistics
Age at initial study visit, years: mean (range)	14.8 (7–21)
Age at diagnosis, years: mean (range)	13.0 (6–17)
Disease duration at initial study visit, years: mean (range)	2.3 (0–15)
Sex	
Male: n (%)	11 (13%)
Female: n (%)	74 (87%)
Race	
Black: n (%)	65 (76%)
White: n (%)	11 (13%)
Asian: n (%)	1 (1%)
Other/Not Specified: n (%)	8 (9%)
Ethnicity	
Hispanic: n (%)	6 (7%)
Not Hispanic: n (%)	79 (93%)
Insurance	
Private: n (%)	45 (52.9%)
Public: n (%)	36 (42.4%)
None: n (%)	4 (4.7%)
Rurality	
Urban (RUCA < 4): n (%)	61 (72%)
Rural (RUCA ≥ 4): n (%)	24 (28%)
SLEDAI-2K score at diagnosis: mean (range)	14.4 (0–38)
SLEDAI-2K score at initial visit: mean (range)	6.2 (0–24)
Number of visits during study timeframe, mean (range)	10.5 (0–33)
Attainment of LLDAS by last visit	
Yes: n (%)	66 (78%)
No: n (%)	19 (22%)
Lupus disease characteristics	
Lupus nephritis: n (% yes)	45 (75%)
CNS lupus: n (% yes)	13 (13%)

RUCA: rural urban commuting area; LLDAS: lupus low disease activity state; CNS: central nervous system.

**Table 2:** Results * of multivariable logistic regression models for binary outcomes among all study participants (n = 85), for state and national multidimensional area-level indicators (MDIs).

	Multidimensional Area-Level Indicator (MDI)		
	SVI Effect Estimate (95% CI) (Higher Vulnerability vs. Lower)	ADI Effect Estimate (95% CI) (Higher Deprivation vs. Lower)	COI Effect Estimate (95%) (Lower Opportunity vs. Higher)
**Models Based on** **State-Ranked MDIs**			
**LLDAS Attainment**	2.92 (0.93, 9.16)	1.70 (0.55, 5.30)	1.29 (0.40, 4.14)
**Lupus Nephritis**	0.92 (0.36, 2.33)	0.84 (0.31, 2.27)	2.21 (0.81, 6.08)
**CNS Lupus**	0.68 (0.18, 2.57)	2.85 (0.60, 13.56)	8.70 (1.30, 58.04) ^†^
**Models Based on Nationally Ranked MDIs**			
**LLDAS Attainment**	4.30 (1.30, 14.29) ^†^	1.82 (0.59, 5.65)	1.11 (0.32, 3.80)
**Lupus Nephritis**	0.76 (0.30, 1.94)	0.95 (0.36, 2.55)	1.97 (0.70, 5.53)
**CNS Lupus**	0.68 (0.18, 2.56)	2.36 (0.52, 10.74)	3.98 (0.69, 23.04)

* Results are expressed as odds ratios and 95% confidence intervals, comparing those with higher vulnerability, higher deprivation, and lower opportunity to those with lower vulnerability, lower deprivation, and higher opportunity, respectively. Covariates in models included age, sex, race/ethnicity, insurance, duration of disease, and length of follow-up time (LLDAS only). Odds ratios greater than one indicate higher odds of attaining outcome among patients living in areas of higher vulnerability/deprivation or lower opportunity (vs. lower vulnerability/deprivation or higher opportunity). ^†^ Statistically significant (*p*-value < 0.05).

## Data Availability

The data presented in this study are available on request from the corresponding author. The data are not publicly available due to privacy and ethical reasons.

## Supplementary Materials

The following supporting information can be downloaded at: https://www.mdpi.com/article/10.3390/children13081062/s1, Supplemental Table S1: Proportions of patients attaining each outcome, stratified by dichotomizations of SVI, ADI, COI, and rurality. All proportions listed are row proportions, meaning that the row-specific ‘N’ is the denominator for a given proportion.; Supplemental Table S2: Results * of multivariable general linear models comparing initial SLEDAI-2K scores by state and national MDIs.

## Abbreviations

The following abbreviations are used in this manuscript:

cSLE	Childhood-Onset Systemic Lupus Erythematosus
MDI	Multidimensional Area-Level Indicator
ADI	Area Deprivation Index
COI	Childhood Opportunity Index
RUCA	Rural–Urban Commuting Area
LLDAS	Low Lupus Disease Activity State
CNS	Central Nervous System
LN	Lupus Nephritis
SLEDAI-2K	Systemic Lupus Erythematosus Disease Activity Index 2000
MUSC	Medical University of South Carolina
CI	Confidence Interval
