# Supplementary material for: Assessing the Relationship Between Multidimensional Area-Level Indicators and Lupus Disease Activity in Children
Source: Children (Basel). 2026 Aug 10;13(8):1062. doi: 10.3390/children13081062 (PMC13511155; doi:10.3390/children13081062)
Supplement: Supplementary file 1 [file children-13-01062-s001.zip › children-4429973-supplementary.pdf]

Supplemental Table S1. Proportions of patients attaining each outcome, stratified by dichotomizations of SVI, ADI, COI, and rurality. All proportions listed are row proportions, meaning that the row-specific 'N' is the denominator for a given proportion.

Metric	Category	N	LLDAS	Lupus Nephritis	CNS Lupus	≥1 SLE-Related ED Visit	≥1 SLE-Related Hospitalization
**SVI (state)**	Less vulnerable (<0.35)	41	70.7%	51.2%	17.1%	19.5%	41.5%
	More vulnerable (≥0.35)	44	84.1%	54.6%	13.6%	27.3%	38.6%
**SVI (national)**	Less vulnerable (<0.65)	41	68.3%	53.7%	17.1%	22.0%	43.9%
	More vulnerable (≥0.65)	44	86.4%	52.3%	13.6%	25.0%	36.4%
**ADI (state)**	Less deprived (<6)	42	76.2%	50.0%	9.5%	16.7%	38.1%
	More deprived (≥6)	41	78.1%	53.7%	17.1%	26.8%	39.0%
**ADI (national)**	Less deprived (<72)	41	75.6%	48.8%	9.8%	17.1%	39.0%
	More deprived (≥72)	42	75.6%	54.8%	16.7%	26.2%	38.1%
**COI (state)**	More opportunity (≥Moderate)	39	79.5%	41.0%	7.1%	20.5%	30.8%
	Less opportunity (<Moderate)	46	76.1%	63.0%	21.7%	26.1%	47.8%
**COI (national)**	More opportunity (≥Moderate)	31	80.7%	38.7%	6.5%	22.6%	29.0%
	Less opportunity (<Moderate)	54	75.9%	61.1%	20.4%	24.1%	46.3%
**Rurality**	Urban	61	75.4%	50.8%	14.8%	21.3%	36.1%
	Rural	24	83.3%	58.3%	16.7%	29.2%	50.0%

SVI: Social Vulnerability Index (range 0 to 1), ADI: Area Deprivation Index, COI: Childhood Opportunity Index
ADI state decile (1-10): 1 (least disadvantaged block group)- 10 (most disadvantaged block group)
ADI national decile (1-100): 1-10 (least disadvantaged block group)- 91-100 (most disadvantaged block group)

Supplement Table S2. Results* of multivariable general linear models comparing initial SLEDAI-2K scores by state and national MDIs

State ranked MDI grouping	N	Mean Estimate (95% CI)	Nationally ranked MDI grouping	N	Mean Estimate (95% CI)
SVI					
< 0.20 (least vulnerable)	17	3.4 (0.2, 6.7)	< 0.54	20	4.6 (1.5, 7.8)
0.2 to < 0.35	24	8.2 (5.6, 10.8)	0.54 to < 0.65	21	8.3 (5.5, 11.0)
0.35 to < 0.65	23	4.6 (1.7, 7.4)	0.65 to < 0.88	23	4.7 (1.8, 7.5)
0.65 to 1.0 (most vulnerable)	21	3.8 (0.6, 6.9)	0.88 to 1.0	21	3.1 (0.0, 6.3)
ADI					
≤ 2nd decile (least deprived)	18	7.1 (3.9, 10.3)	< 48th percentile (least deprived)	20	7.2 (4.1, 10.3)
3rd-5th decile	24	7.0 (4.3, 9.7)	49th-71st percentile	21	7.0 (4.2, 9.8)
6th-8th decile	24	3.4 (1.07, 7.3)	72nd-89th percentile	21	4.0 (0.8, 7.2)
9th-10th decile (most deprived)	17	3.2 (0.0, 6.6)	90th-99th percentile (most deprived)	21	2.9 (0.0, 6.0)
COI					
Very Low (lower opportunity)	30	4.0 (1.0, 7.0)	Very Low (lower opportunity)	33	3.8 (0.8, 6.8)
Low	16	7.2 (3.8, 10.5)	Low	21	6.7 (3.7, 9.7)
Moderate	17	4.0 (0.7, 7.3)	Moderate	17	5.3 (2.1, 8.5)
High	12	7.5 (4.0, 11.0)	High/Very High (higher opportunity)†	14	6.5 (2.9, 10.0)
Very High (higher opportunity)	10	4.9 (0.6, 9.1)			

* Results are expressed as model-adjusted means and 95% CIs, where covariates included age, sex, race/ethnicity, insurance, and duration of disease.

† Since only n=1 participant fell in the 'very high' category, the 'high' and 'very high' categories were combined.
